# UAV Photogrammetry for Estimating Stand Parameters of an Old Japanese Larch Plantation Using Different Filtering Methods at Two Flight Altitudes

**DOI:** 10.3390/s23249907

**Published:** 2023-12-18

**Authors:** Jeyavanan Karthigesu, Toshiaki Owari, Satoshi Tsuyuki, Takuya Hiroshima

**Affiliations:** 1 Department of Global Agricultural Sciences, Graduate School of Agricultural and Life Sciences, The University of Tokyo, Tokyo 113-8657, Japan; karthigesu-jeyavanan543@g.ecc.u-tokyo.ac.jp (J.K.); tsuyuki@fr.a.u-tokyo.ac.jp (S.T.); hiroshim@g.ecc.u-tokyo.ac.jp (T.H.); 2 Department of Agronomy, Faculty of Agriculture, University of Jaffna, Jaffna 40000, Sri Lanka; 3 The University of Tokyo Hokkaido Forest, Graduate School of Agricultural and Life Sciences, The University of Tokyo, Furano 079-1563, Hokkaido, Japan

**Keywords:** old-aged plantation, Japanese larch, UAV photogrammetry, stand parameters

## Abstract

Old plantations are iconic sites, and estimating stand parameters is crucial for valuation and management. This study aimed to estimate stand parameters of a 115-year-old Japanese larch (*Larix kaempferi* (Lamb.) Carrière) plantation at the University of Tokyo Hokkaido Forest (UTHF) in central Hokkaido, northern Japan, using unmanned aerial vehicle (UAV) photogrammetry. High-resolution RGB imagery was collected using a DJI Matrice 300 real-time kinematic (RTK) at altitudes of 80 and 120 m. Structure from motion (SfM) technology was applied to generate 3D point clouds and orthomosaics. We used different filtering methods, search radii, and window sizes for individual tree detection (ITD), and tree height (TH) and crown area (CA) were estimated from a canopy height model (CHM). Additionally, a freely available shiny R package (SRP) and manually digitalized CA were used. A multiple linear regression (MLR) model was used to estimate the diameter at breast height (DBH), stem volume (V), and carbon stock (CST). Higher accuracy was obtained for ITD (F-score: 0.8–0.87) and TH (R^2^: 0.76–0.77; RMSE: 1.45–1.55 m) than for other stand parameters. Overall, the flying altitude of the UAV and selected filtering methods influenced the success of stand parameter estimation in old-aged plantations, with the UAV at 80 m generating more accurate results for ITD, CA, and DBH, while the UAV at 120 m produced higher accuracy for TH, V, and CST with Gaussian and mean filtering.

## 1. Introduction

Old plantations are iconic sites that have great value. Assessing stand parameters at old plantations is therefore extremely important [1]. The Japanese larch (*Larix kaempferi* (Lamb.) Carrière) is an endemic coniferous species in Honshu Island, central Japan [2,3,4,5], but it is a non-native and key plantation species in Hokkaido, northern Japan [6]. It is an economically important deciduous conifer species that grows in cool-temperate forests [4]. Japanese larch has suitable characteristics for forestry, and plantations were introduced to Hokkaido from the central mountainous region of Honshu in the early part of the last century [3]. These plantations have succeeded due to their rapid growth and disease- and cold-resistance characteristics compared to other planting species. The Japanese larch was therefore extensively used for reforestation in northern Japan from 1960 to the 1970s [7]. According to the Forestry Agency, in the National Forest Inventory of Japan, only 3% of the total forest area consists of larch (including both natural and plantation forests), whereas its proportion in Japan’s planted forests is 10% [8]. The recommended long-cutting period of Japanese larch is 40–60 years [9,10]. We considered our study site to be an old plantation because it was more than 100 years old, i.e., approximately double the age of the cutting period. However, stand parameter data for old larch plantations are scarce in the region.

Forest inventory information is extremely important for forest management. The tree height (TH) and diameter at breast height (DBH) provide useful information in the field of forest research, allowing for the quantification of timber resources, evaluation of the ecological and economic value of the forest stand, computation of the number of individual trees, stem volume (V), and carbon stock (CST), and understanding the rate and pattern of forest regeneration [11,12,13]. In forest management, TH and crown area (CA) are used to develop the allometric equations for CST calculation and a broad range of stand attributes [14,15]. For example, TH was used for both individual tree volume and stand volume estimation. The estimated volume in the forest is important for the assessment of the hydrological cycle [16], and the production capacity of the site and a single tree [17]. Further, stand density, competition, and survival are characterized by CA [18,19]. Stand density is a significant parameter that explains the dimension and distribution of trees [20]. 

The collection of field data is laborious, time-consuming, and only appropriate for small forest stands. High accuracy in stand parameter estimation is difficult when using remote sensing technology due to issues related to uncertainty, technology, the availability of high-spatial-resolution data, and cost. Light detection and ranging (LiDAR) provides accurate data but is not suitable for large-scale forest monitoring due to its high cost. Satellite data can be very affordable; however, there are several limiting factors such as a relatively low spatial resolution, occlusion by cloud cover, and difficulties in obtaining them at specific times. Unmanned aerial vehicle (UAV) technology is a recent advance in remote sensing that can be used to characterize plantation forest [21]. UAVs are the most efficient platforms for obtaining remotely sensed data and can provide extremely high spatial resolution, low-cost data, and cloud-free images with high versatility, flexibility, and adaptability [22]. The use of UAV photogrammetry provides high-resolution images for estimating individual tree position, and TH, and for crown delineation, with high accuracy [14,23]. However, digital terrain models (DTMs) generated from UAV photogrammetry lack accuracy due to the occlusion effect [24,25,26]. It has been reported that UAV photogrammetry during leaf-off conditions is able to generate an accurate UAV DTM [27]. Moe et al. [28] achieved a high accuracy (63–73%) for forest canopy classification in a complex mixed conifer–broadleaf forest using the combination of a UAV digital surface model (DSM) and airborne LiDAR DTM. Wang et al. [27] used a radial basis function neural network (RBFNN) with spatial interpolation to achieve high accuracy in a UAV DSM. Hastaoglu et al. [29] used an inverse distance weighted model (IDW) that took into account field slope and directional distributions of reference points in IDW-based interpolations to increase the accuracy of DTM. However, in a dense forest, it is not possible to derive an accurate DTM using photogrammetric methods, because insufficient ground surface is visible in the aerial images [24,25,26]. Xu et al. [30] built a high-precision DTM from the point cloud generated by LiDAR and then subtracted the DTM from the DSM generated from the photogrammetric point cloud to obtain the CHM.

Tree crowns and other structural variables are extracted either from canopy height models (CHMs) or normalized point clouds for individual tree detection (ITD). Individual tree metrics are extracted within the segmented tree crowns. However, tree density, forest type, and tree species are the main factors influencing the accuracy of tree crown detection [31,32]. Even though satisfactory results were obtained for conifer plantations in many studies [33,34], stand parameter estimation in old Sugi (*Cryptomeria japonica*), Hinoki (*Chamaecyparis obtusa*), and other conifer plantations was performed using UAV technology [35,36,37,38]. However, there have been no studies of old larch plantations using UAV technology. Many algorithms have been used to distinguish tree crowns, namely, inverse watershed segmentation (IWS), watershed segmentation (WS), seed growing segmentation (SG), and object-based image segmentation (OBIA) [39,40]. In addition, various smoothing techniques, such as lowpass, highpass, Gaussian, and mean filtering, at different kernel sizes, have been used in many studies. Improvements in the quality of remote sensing data and processing workflows have recently enabled remote forest mapping to become more analogous to field-based approaches that involve detecting and characterizing individual trees [41,42,43]. Nasiri et al. [44] found that lowpass filtering with a circular neighborhood at a 25-cell radius (kernel size; cell size with respect to the size of the largest area within the scene) provided highly accurate ITD. Similarly, various software packages have been used for crown segmentation, such as eCognition (Trimble Inc., Sunnyvale, CA, USA), Labkit (in Fiji) [45], and ArcGIS (ESRI, Redlands, CA, USA). In QGIS (Open–Source Geospatial Foundation), the System for Automated Geoscientific Analyses (SAGA) software (version 2.1.4) is used to conduct the segmentation process. The WS approach has been shown to have an acceptable ability to delineate tree crowns using a CHM in a closed forest canopy structure [14,46]. Moe et al. [28] studied crown segmentation using OBIA and a multiresolution segmentation algorithm using the eCognition Developer, and accuracy was confirmed by manual delineation of crown cover (CC). Different algorithms give different tree crown diameters for different flight altitudes [40]. 

In this study, we examined the capabilities of high-resolution UAV imagery to estimate ITD, TH, CA, DBH, V, and CST using various filtering methods, and flight altitudes of 80 and 120 m, in an old larch plantation site. We considered the following questions: Can a UAV generate an accurate CHM and high-resolution orthomosaic in an old larch plantation? How do different UAV flying altitudes and filtering methods improve ITD? Can UAV photogrammetry estimate TH accurately in an old larch plantation? Can UAV photogrammetry estimate the CA and CC? What are the most important UAV-derived metrics for estimating DBH, V, and CST? To estimate these stand parameters, we used various filtering methods, i.e., lowpass, Gaussian, and mean, at different search radii and window sizes using a combination of ArcGIS Pro and QGIS in the SAGA and the open-source shiny R package (SRP) [47,48].

## 2. Review of Literature

Photogrammetry is a technique that derives the required information by creating a 3D model from 2D images. Common points are matched from a series of overlapping 2D images to create the 3D model through Structure-from-Motion (SfM) technology [49,50,51]. The photogrammetry technique has been applied in many fields such as surveying, civil engineering, urban planning, gas detection, fire monitoring, archeology, mining, industry, urban management, agriculture, and forest management [49,51,52].

In the sustainable forest management approach, the estimation of forest stand parameters is extremely important. Gómez et al. [53] stated that age class, stem density, stem frequency, DBH, CA, crown closure, mean crown size, crown width, circumference, TH, mean stand height, maximum height, basal area, biomass, and stand V are forest structural parameters estimated in many studies using high spatial resolution (HSR) satellite imagery (IKONOS, Pan, Pan-sharpened, QuickBird, and SPOT). Gómez et al. [53] extracted quadratic mean diameter, basal area, and tree density as forest structural parameters to assess wood volume and biomass using QuickBird-2 imagery. Spatial resolution is an important consideration when using remote sensing for forest characterization [54]. In addition to the use of HSR satellite imagery, recent advances in UAVs have provided high-resolution imagery, enabling more reliable forest structure estimates with high accuracy. Jayathunga et al. [55] estimated the standard deviation of height, percentile height, coefficient of variation in height, skewness, and kurtosis, and canopy cover above mean height using Fixed-Wing UAV. Gao et al. [56] combined UAV laser scanning and ground backpack laser sacking to extract individual tree structural parameters and fit volume models in subtropical planted forests in southeastern China.

Belmonte et al. [57] found that estimates of individual tree height and crown diameter were most accurate at low stand density, with significantly reduced accuracy at high stand density using UAV photogrammetry. Individual DBH and stand-level estimates of basal area, stand density, and canopy cover (CC) are commonly used as forest mensuration metrics. Kameyama and Sugiura [58] estimated the CA and TH using different SfM software such as Terra Mapper (version 2.5.1), PhotoScan (version 1.3.2.4205), and Pix4Dmapper (version 4.5.6) on aerial image processing by UAV at different altitudes. The UAV has been used successfully in several recent studies to predict the DBH distribution of trees [59], mean TH [60], and aboveground CST [10]. In addition, the high point density of UAV data allows the crowns of individual trees to be delineated. This improves the accuracy of the ITD [61]. The SRP is a freely available application developed with a LiDAR analysis tool and a standalone R package called treetop. The treetop package is publicly available on the SRP, which is a service platform for hosting shiny web apps (https://carlosasilva.shinyapps.io/weblidar-treetop/, accessed on 10 February 2023). Detailed methods for its application are provided by Silva et al. [47,62]. The treetop package is capable of fast and effective ITD and crown delineation and is also applicable to UAV-derived CHM [47]. The local maximum (LM) is an algorithm that finds a maximum height in the CHM that indicates treetops [63]. The treetops and CA were extracted automatically by adjusting the two types of window sizes, referred to as a smoothing window size (SWS), and a fixed window size (FWS). The spurious local maxima detected in the CHM are eliminated by the application of a smoothing filter, which will increase tree detection accuracy [64]. Voronoi tessellation-based algorithm is particularly suitable for dense areas of conifer [65] and broadleaf forests [66]. The Voronoi tessellation algorithm was considered suitable for our study area due to the dense canopy of the old larch plantation. Moe et al. [28] visually interpreted the orthomosaic to digitize the conifer tree crown due to the absence of field data for CA and reported that the manual CA had high accuracy compared with the field CA. Mohan et al. [67] visually interpreted high-resolution imagery.

DBH measurement in the field gives an accurate estimation and it is highly correlated with other tree parameters [68]. DBH is used as a predictor variable to develop the stem V equations, tree growth model, and biomass equations. LiDAR data have been used in many studies for the estimation of individual tree DBH [60,68,69]. Liang et al. [70] stated that trunk position and DBH accuracy of individual trees were 88.2% and 90.4%, respectively, using the SfM point cloud. Piermattei et al. [71] found that the tree detection rate and bias of the extracted DBH were 69–98% and 1.13 cm, respectively, using SfM point clouds. Sun et al. [72] applied different methods such as the linear regression model, a linear model with ridge regularization, support vector regression, random forest, artificial neural network, and k-nearest neighbors to predict the individual DBH of Larch (*Larix olgensis*) using UAV-LiDAR. They reported that all methods improved the accuracy of the predictions except linear regression.

In old-growth forests, stand parameters are applied on a local to regional scale using detailed data, often from airborne laser scanning [73]. ITD, TH, CA, lying deadwood, standing deadwood, canopy cover, stand height, stand density, dominant height, height distribution, gap detection, aboveground biomass, timber V, and tree species were estimated using airborne laser scanning, optical very high-resolution, and synthetic aperture radar in old-growth forest. Qiu et al. [74] estimated the TH, DBH, crown width, and age in an old pear orchard using UAV photogrammetry and obtained an RMSE of 0.1814 m, 3.0039 cm, 0.3292 m, and 4.3753 years, respectively. Holiakaa et al. [75] used UAV photogrammetry to estimate ITD, TH, and biomass in different ages of a Scots pine forest, including a 115-year-old stand. Zhou and Zhang [76] estimated TH, CA, and biomass of larch (*Larix gmelinii*) and Chinese pine (*Pinus tabuliformis*) plantations of different ages using UAV oblique photogrammetry. Although many previous studies have also demonstrated the great potential of UAVs for estimating forest structural parameters and their advantages over airborne LiDAR, the use of UAV photogrammetry with RGB imagery in old larch plantations has not been fully explored.

## 3. Materials and Methods

### 3.1. Study Site

**Figure 1:**
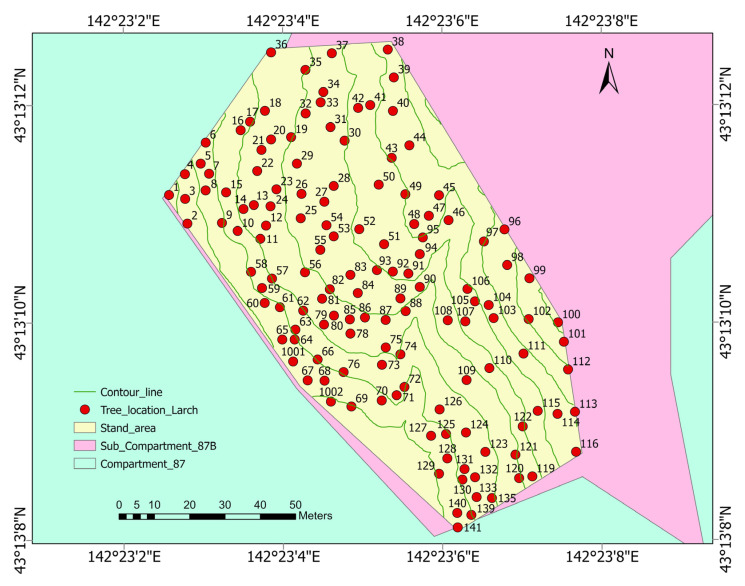
The study area map (Coordinate system: JGD2000 Japan–19 zone XII/GSIGEO 2000 geoid): Study locations (43°13′ N, 142°23′ E) of the larch plantation in the forest management sub-compartment 87B in the University of Tokyo Hokkaido Forest (UTHF) in Japan. The red dot with value represents the spatial position of a larch tree with tree number. Green, pink and yellow areas represent compartment 87, sub compartment 87B and larch stand area, respectively.

Figure 1 is a map of the study area. A 115-year-old Japanese larch plantation site was selected (43°12′55″ N, 142°23′7″ E, 43°13′8″ N, 142°23′31″ E) at the University of Tokyo Hokkaido Forest (UTHF) in Furano City, on Hokkaido Island in northern Japan [77]. The site was planted in 1908 with a seedling density of 3000 stem ha^−1^. The larch plantation is located in sub-compartment 87B of UTHF. The study area extends for 0.93 ha with a mean temperature: 6.6 °C and precipitation of 1196 mm/year at the arboretum (230 m). Snow covers the ground from late November to early April, with a maximum depth of approximately 1 m. The elevation is 250–300 m above sea level and the slope is 18–20°.

### 3.2. Field Data

A field survey was conducted in November 2022. A total of 136 individual larch trees were measured. Seven other individual trees of other species were identified in the forest but were not sampled. Tree spatial position, TH, and DBH (1.3 m above ground) were measured. The TH was measured using a Vertex III hypsometer and transponder (Haglöf Sweden AB, Långsele, Sweden). Tree DBH was measured using diameter tape. The tree spatial locations were measured in 2007 using an Impulse laser rangefinder with a Mapstar electronic compass module (Laser Technology, Inc., Centennial, CO, USA). The ITD, basal area (BA), V, and CST were calculated from these field-measured parameters. We used a species-specific volume table provided by UTHF. The CST was calculated using the following allometric Equation (1) [78]:

(1) $$CST=\sum_{j}\left\{\left[V_{j}\times D_{j}\times {BEF}_{j}\right]\times \left(1+R_{j}\right)\times CF\right\}$$

where CST is the carbon stock in living biomass (MgC ha^–1^); V is the merchantable volume (m^3^ ha^–1^), it is a volume estimated for each tree species based on the yield table developed for a given region, site class, and stand age; D is the wood density (t–d.m. m^–3^); BEF is the biomass expansion factor for the conversion of volume; R is the root-to-shoot ratio; CF is the carbon fraction of dry matter (MgC t–d.m.^–1^); and j is the tree species [78]. For species larch, the value of D, BEF, R, and CF was 0.404, 1.15, 0.29, and 0.51, respectively, as suggested by Greenhouse Gas Inventory Office of Japan and Ministry of Environment, Japan [78].

The summary statistics of the field data are given in Table 1. The tree density, stand volume, and CST of the larch trees in the stand were 147 stems ha^–1^, 543 m^3^ ha^−1^, and 168 MgC ha^−1^, respectively. 

### 3.3. UAV Data

**Figure 2:**
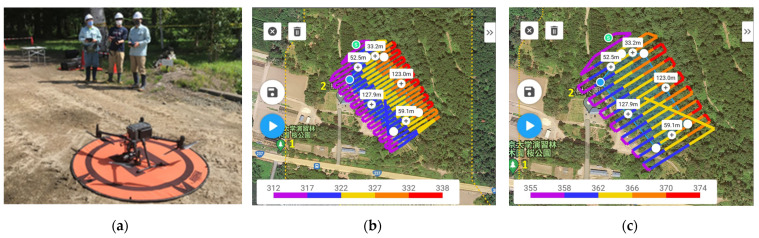
UAV photogrammetry process in the field: (**a**) DJI M300 RTK UAV in the study area; (**b**) UAV flight plan at 80 m altitude with the base map; (**c**) UAV flight plan at 120 m altitude with the base map. Legend of different colors shows the elevation of the study area from mean sea level whereas the plus (+) sign with a value represents the length of one side of the flight area in the respective locations. In this case, there were five numbers due to the pentagon shape of the flight plan. The actual flight path had a buffer of 30 m around the flight area. Symbols 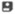, 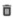, 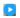, 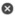 represents the save (tap to save current settings and create a mission flight), delete selected waypoint (tap to delete the selected waypoint), start flight button (tap to perform the flight mission), and clear waypoints (tap to clear all the added waypoint), respectively. The meaning of location 1. 東京大学樹木園桜公園 and 2. 樹木園 are the cherry blossom park of the University of Tokyo arboretum and arboretum, respectively.

**Figure A1:**
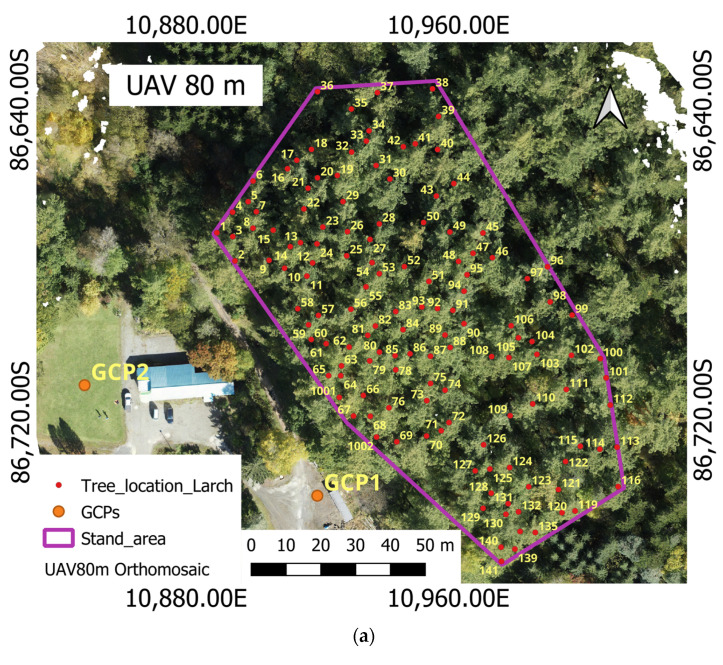

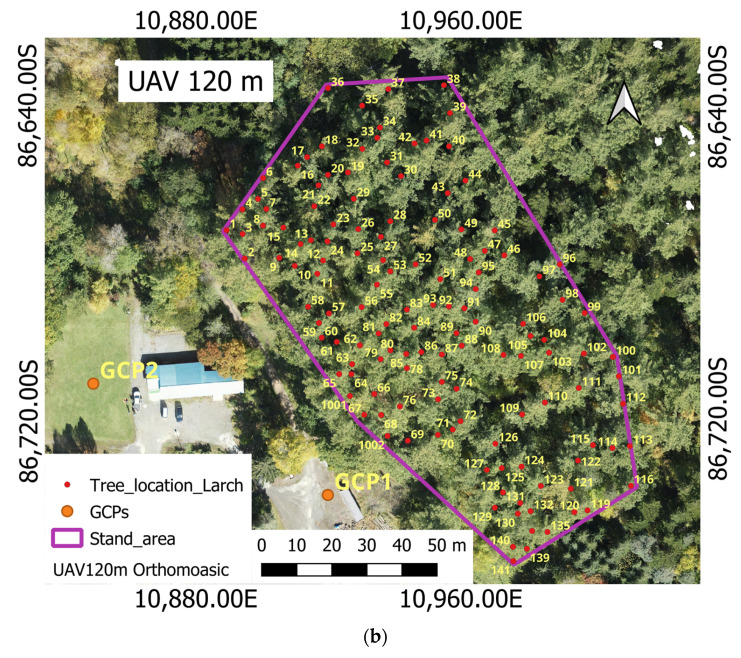
Representation of the field tree location in the respective orthomosaics: (**a**) Field tree location and stand area in derived orthomosaic at UAV 80 m; (**b**) Field tree location and stand area in derived orthomosaic at UAV 120 m. Red dots indicate the spatial location of the trees while numbers (yellow color) represent the respective tree number labeled in the field. Two orange dots represent the distribution of the ground control points, GCP 1 and GCP 2.

The UAV image collection process and the parameter settings in the field are given in Figure 2 and Table 2, respectively. The UAV imagery was acquired using a Matrice 300 real-time kinematic (RTK) drone with a Zenmuse P1 sensor (DJI, Shenzhen, China) on 13 October 2022. The front and side overlap were both 90%. Flight planning was performed using DJI Pilot2 software (version 6.1.2), and the location details were sent to the UAV drone in the field. Two ground control points (GCPs) were used (Figure A1). The GCPs, take-off, and landing points were set in available open areas before the flight missions [77]. The xyz coordinates of the GCPs were recorded with an RTK global satellite navigation system (GNSS) receiver (DG-PRO1RWS, BizStation Corp., Tokyo, Japan), with a positional accuracy of <0.02 m. Two batteries were required for a one-time flight of approximately 30 min, which was less than the theoretical time (55 min) due to the environmental conditions and time allocation for a flight return to the station. The flight missions proceeded at flight heights of 80 m (UAV 80 m) and 120 m (UAV 120 m). The terrain following mode was selected in the settings of the UAV flight missions. 

### 3.4. Data Analysis

#### 3.4.1. UAV Image Processing

**Figure 3:**
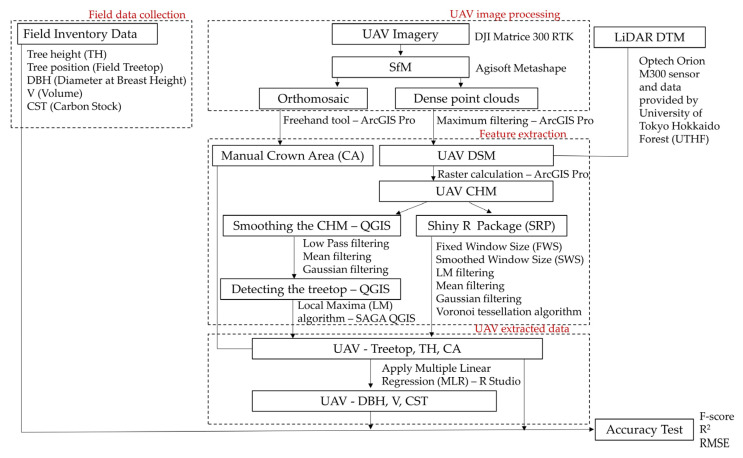
The workflow of the study including field data collection, UAV photogrammetry process; Canopy height model (CHM) generation; Feature extraction—Treetop, TH, and CA; manual CA delineation process; DBH, V, and CST estimation; and accuracy test.

The overall workflow of the study is shown in Figure 3. The professional photogrammetric processing software Agisoft Metashape 1.8.4 (Agisoft LLC, St. Petersburg, Russia) was used for UAV image processing. The parameter settings for UAV image processing are given in Table A1. Image alignment, building a dense point cloud, building a digital elevation model (DEM), and building an orthomosaic were processed. Medium accuracy was set to optimize the camera location, orientation, and other internal parameters during the image alignment and building of dense point cloud stages, to reduce the processing time (Table A2). Image processing was performed separately for UAV 80 m and UAV 120 m.

The GCPs were added to each corresponding image for optimization of the camera locations and orientations, as well as other internal camera parameters. The depth filtering mode during the photogrammetric process is considered to remove noticeable outliers while preserving as much as possible the detailed elements of the three-dimensional model [79]. Tavasci et al. [79], Moe et al. [77], and Jayathunga et al. [15] used mild depth filtering for the automated removal of outliers. We also used mild depth filtering, which was set to remove outliers. The Tokyo Japan plane rectangular CS XII (ESPG: 2454) coordinate system was used for georeferencing. We followed the Agisoft Metashape default setting for the DEM building stage and orthomosaic building stage. Orthomosaics were exported in GeoTiff format, and dense point clouds were exported in LAS format.

#### 3.4.2. Generation of the CHM

The LAS files of the 3D point clouds generated by Agisoft Metashape were used to generate the DSM. The LAS files were input to ArcGIS Pro (version 2.8) for DSM generation. First, an LAS dataset was used as input to ArcGIS. Then, a raster was created using the LAS dataset [80], with the file value set to elevation. For DSM generation, the LAS file was filtered to the first return, and the value was set to maximum. A binning approach was adopted, with values assigned to the nearest cell using the void fill function as a natural neighbor technique. The UAV DTM was not accurate due to the occlusion effect of the top canopy. LiDAR can penetrate the forest canopy to the interior and the ground through laser echoes, thereby obtaining vertical forest structure information and enabling the generation of a high-precision DTM. Therefore, we generated the CHMs for respective UAV flights using each pixel value of the UAV DSM by subtracting the LiDAR DTM from the UAV DSM. We used the LiDAR DTM generated in 2018 by UTHF using an Optech Airborne Laser Terrain Mapper (ALTM) Orion M300 sensor (Teledyne Technologies, Thousand Oaks, CA, USA) mounted on a helicopter that flew 600 m aboveground at a speed of 140.4 km h^–1^. The course overlap, pulse rate, scan angle, beam divergence, and point density of LiDAR data were 50%, 100 kHz, ±20°, 0.16 mrad, and 11.6 points per m^2^, respectively. The flight was designed to optimize image overlap and distribution, using high-resolution imagery across the survey area to generate a dense and accurate point cloud. The GCPs were measured using RTK GNSS for precise measurement and the coordinates (latitude, longitude, and elevation) were recorded. Classified LiDAR point data, e.g., ground, non-ground, first, second, third, and last returns, were delivered by the data provider (Hokkaido Aero Asahi, Hokkaido, Japan) and data were stored in LAS format. The DTM was generated from LiDAR point clouds using well-distributed GCPs (seven checkpoints) spatially and covered a representative portion of the terrain all over the UTHF. The LiDAR ground returns were used to develop a digital terrain model (LiDAR-DTM) [77]. Minimum height, maximum height, average height, RMSE, and standard deviation of the derived LiDAR DTM were 0.02 m, 0.14 m, 0.00 m, 0.061 m, and 0.061 m, respectively. Both average height and standard deviation were below the limit of 0.25 m (Work Regulations Article 326-3, Hokkaido Aero Asahi, Hokkaido, Japan), confirming that the local elevation and laser elevation were consistent. In our study, the stand area of the LiDAR DTM was clipped to generate the CHM.

#### 3.4.3. Individual Tree Detection

The UAV ITD approach used a combination of filtering, search radius, and window sizes, as shown in Table 3. The CHM was used as the input for ITD. Various filtering methods were used, such as lowpass, Gaussian, and mean. Different search radii/sigma, window sizes, and circular searches were used to achieve highly accurate tree detection. A local minima and maxima algorithm in QGIS SAGA was used to identify individual treetops in the filtered CHM. We also used an SRP for treetop detection, TH, and crown delineation [47,62]. In our study, we performed the ITD with the SRP using two window sizes, FWS = 3 × 3 and 5 × 5 and SWS = 3 × 3 and 5 × 5, where the maximum crown factor and exclusion parameters were set to 0.4 and 0.7, respectively. The TH threshold used was 1.37 with 0.5 m resolution in the CHM as a default setting (see the shiny web apps for more detail).

The LM algorithm identified the field treetop as the UAV treetop when their locations were similar or identical. These cases were taken to indicate correctly detected trees (true positive; TP). When the field treetop and UAV treetop were found in the CHM of a respective tree crown, it was classified as TP. We also validated the detected trees when the location of the field treetop matched the UAV treetop in the CHM. When there was no UAV treetop close to the field treetop in the CHM of a tree crown, it was taken to indicate incorrectly undetected trees (false negative; FN). Sometimes, the UAV treetop was close to the field treetop of another tree but not close to the CHM of the respective field tree, which was considered to represent an incorrectly undetected tree. Cases where there was no field treetop, but the UAV treetop was found in the CHM, were taken to indicate incorrectly detected trees (false positive; FP). Cases where there was no field treetop and no UAV treetop in the CHM were classified as correctly undetected (true negative; TN). The distance threshold for searching field treetops neighboring the UAV treetops was the search radius or sigma, based on the window sizes in the filtered or smoothed CHM. 

#### 3.4.4. Tree Height Estimation

The generated CHM was used to extract the TH using different filtering methods. The CHM was clipped within the stand area using the Extract by Mask function [81] in ArcGIS Pro to extract the individual TH using different algorithms. The UAV tree locations were identified by the LM algorithm with lowpass, mean, and Gaussian filtering. First, UAV treetops were converted to raster data using the point-to-raster function to make them compatible for extraction. Then, they were input to the CHM, and the spatial location UAV treetops were used as input raster or feature mask data. Then, the extracted raster data of UAV treetops were converted from raster to point data to obtain the TH attribute table. The summary statistics function was used to derive the mean, minimum, maximum, standard deviation, and variance for the respective UAV TH. We also derived the TH information from the SRP, which automatically generated the TH [47].

#### 3.4.5. Tree Crown Delineation

The UAV orthomosaic was used to manually digitalize the CA because of its high resolution. First, a new shape file was created, and the respective tree crown was then manually delineated using a freehand tool on the newly created shape file while viewing the orthomosaic in ArcGIS Pro. Then, CA was determined using a geometric calculation. We also used the SRP to derive the CA. We set the parameters by adjusting the window sizes (Table 3). As FWS increased, the number of trees detected decreased [47,82]. Once individual treetops were detected, their crown boundaries were delineated using the Voronoi tessellation-based algorithm developed by Silva et al. [62]. This algorithm was operated with the LM algorithm and used the maximum crown factor and exclusion parameters, both ranging from 0 to 1, to define the crown boundaries on the UAV CHM, delimiting the boundary of the grid cells belonging to each tree. In this study, we used the CA derived from manual digitalization and the SRP.

#### 3.4.6. Tree DBH, V, and CST Estimation

Individual tree parameters were estimated between segmented tree crowns and manually digitized polygons using one-to-one relationships in previous studies [37]. Moe et al. [28] extracted individual tree parameters from the manually delineated tree crowns using LiDAR and UAV-DAP-normalized point clouds in fusion software. In this study, we used the RGB imagery generated from UAV point clouds in the ArcGIS Pro software (version 2.8) package and SRP to derive the structural variables. The dependent variables of tree DBH, V, and CST were modeled with independent variables such as the manual CA, tree crown perimeter (C_peri), near distance (ND), SRP CA, and UAV flight height derived from the lowpass, mean, and Gaussian filtering methods. 

#### 3.4.7. Accuracy Assessment and Validation

For accuracy assessment and validation, the parameters extracted from the UVA were compared with the field-measured parameters of the larch trees. The seven trees of other species found in the larch plantation were excluded from the analysis.

The F-score was calculated for ITD. The F-score is based on the harmonic mean of precision and recall. The evaluation produced three types of segmentation results. If a tree existed and was identified successfully, it was labeled TP, representing correct segmentation. Under-segmentation is represented; if a tree existed but was not detected, it was labeled FN [83]. Similarly, over-segmentation is represented; if a tree was detected, but did not exist on the ground, it was labeled FP. The overall accuracy of the individual tree detection is calculated using F-score based on precision and accuracy [84,85]:

(2) $$Precision=\frac{TP}{TP+FP}$$

(3) $$Recall=\frac{TP}{TP+FN}$$

(4) $$F\text{-}score=2\times \frac{Precision\times Recall}{Precision+Recall}$$

where precision represents detection accuracy (commission) (2); recall represents detection rate (omission) (3); and the F-score is the weighted average taking both detection rate and detection accuracy into consideration (4).

For the regression of TH and CA, a simple linear regression model was used. The most common methods used for statistical analysis and validation of ground data are the root mean square error (RMSE) (5) and coefficient of determination (R^2^) (6) [11].

(5) $$RMSE=\sqrt{\frac{1}{N}\sum_{i=1}^{N}{{(y}_{i}-\hat{y})}^{2}}$$

(6) $$R^{2}=1-\frac{\sum ({y_{i}-\hat{y})}^{2}}{\sum {{(y}_{i}-\overset{\text{-}}{y})}^{2}}$$

where ŷ= predicted value of y; ȳ = mean value of y.

For tree DBH, V, and CST, a multiple linear regression (MLR) model was used (7) using R studio version 4.22. The final models were selected based on Akaike’s information criterion (AIC) and stepwise variable selection for UAV-derived stand parameters [86] in which the variables with a variance inflation factor (VIF) > 5 were removed to avoid multicollinearity [87]. The leave-one-out-cross-validation method was used to validate the accuracy of the selected models.

(7) $$y=\beta_{0}+\beta_{1}X_{1}+\beta_{2}X_{2}+\ldots \ldots +\beta_{n}X_{n}+\epsilon$$

where y = the predicted value of the dependent variable (DBH, V or CST); $\beta_{0}=$ the y-intercept; independent variables were UAV TH, manual CA, UAV CA, tree crown perimeter (C_peri), and near distance (ND) at respective UAV flight altitude. $\beta_{1}=$ the regression coefficient ($\beta_{1}$) of the first independent variable ($X_{1}$); $\beta_{2}X_{2}$ = the regression coefficient ($\beta_{2}$) of the second independent variable ($X_{2}$); $\beta_{n}=$ the regression coefficient of the last independent variable; and ϵ = model error. 

## 4. Results

### 4.1. The CHM and Orthomosaic

**Figure 4:**
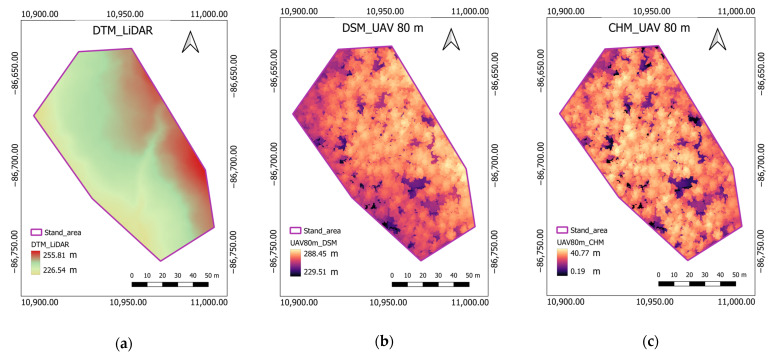

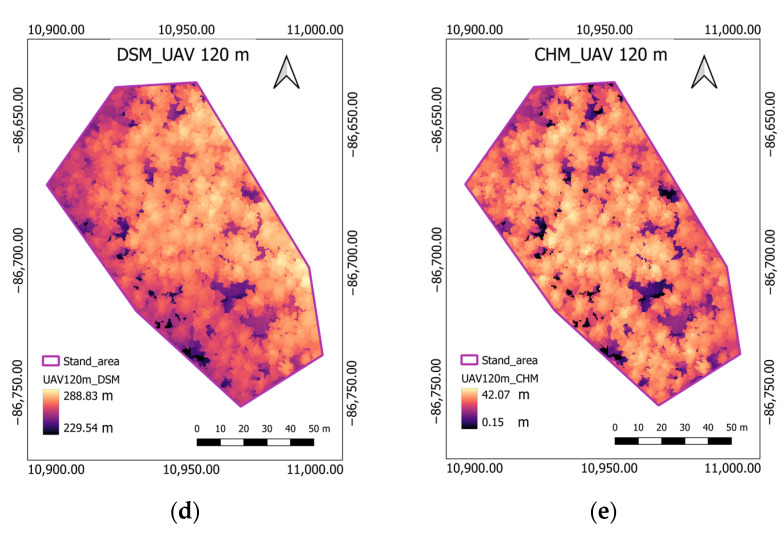
The illustration of CHM derivation from UAV DSM and LiDAR DTM at two flight altitudes, UAV 80 m and UAV 120 m: (**a**) LiDAR DTM; (**b**) UAV DSM 80 m; (**c**) UAV CHM 80 m; (**d**) UAV DSM 120 m; (**e**) UAV CHM 120 m.

The CHM was generated at two flight altitudes from the respective UAV DSM and LiDAR DTM (Figure 4), and orthomosaics were created in which tree locations and the stand area were represented (Figure A1). The maximum TH in the CHMs was 40.77 m for UAV 80 m (Figure 4c) and 42.07 m for UAV 120 m (Figure 4e), whereas the field maximum TH was 42.90 m (Table 1). The maximum TH in both CHMs was lower than the field tree maximum. We generated a high-resolution orthomosaic with a pixel value of 3.25 cm/pix for UAV 80 m and 5.39 cm/pix for UAV 120 m, in which the tree crowns were clearly visible. The total number of images was 1257 and 342 for UAV 80 m and UAV 120 m, respectively. All images had a resolution of 8192 × 5460 pixels (Table A3). 

### 4.2. Individual Tree Detection and Tree Density

**Figure A2:**
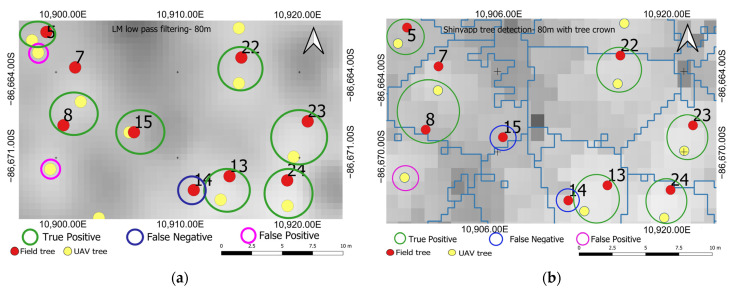
Representation of UAV treetop and field treetop for part of the stand, in which illustration of TP, FN, and FP: (**a**) LM treetop (**b**) SRP treetop. Where TP—is the number of correctly detected trees; FP—is the number of incorrectly detected trees; FN—is the number of incorrectly undetected trees; TN—not applicable, is denoted as those places where no tree exists and the model finds no trees.

The ITD results are given in Table 4, and representations of UAV treetops and field treetops for part of the stand are shown in Figure A2. A total of 143 individual trees were identified in the field, including 136 larch trees and seven other trees within the stand area. Five *Pinus nigra* Arnold, one *Kalopanax septemlobus* (Thunb.) Koidz, and one *Abies sachalinensis* (F. Schmidt) Mast. were identified as the other species in the stand. We only considered larch trees for the ITD and other stand parameter estimations. For ITD, more UAV treetops were identified by LM lowpass (LML) filtering than LM Gaussian (LMG) filtering. For both methods, UAV treetops were significantly (*p* < 0.0001) higher than field treetops. The number of UAV treetops was reduced when the search radius was increased. The LM algorithm detected field treetops at a threshold search radius of 2 m in the LML filtering. The optimum number of UAV treetops in LML filtering was 176 at UAV 80 m and 178 at UAV 120 m. We also compared these results with those of treetop detection with the SRP. The SRP treetop detection performance was higher at the combination of 5 × 5 FWS and 3 × 3 SWS than at the combination of 3 × 3 FWS and 3 × 3 SWS, for mean and Gaussian filtering at threshold search radii of 5 and 2 m, respectively. When the FWS or SMS decreased, SRP detected more treetops than field treetops. For the UAV 80 m flight, ITD was 144 by shiny mean (SM) filtering and 145 by shiny Gaussian (SG) filtering. For the UAV 120 m flight, ITD was 142 by both the SM and SG filtering methods.

The F-score for ITD ranged from 0.76 to 0.87. This indicated that both LML filtering and the SRP performed well in detecting the treetop. However, the F-score was higher for the SRP (0.87 at UAV 80 m and 0.83 at UAV 120 m) than LML filtering (0.79 at UAV 80 m and 0.76 at UAV 120 m). The UAV treetops detected by LM filtering were higher than those detected with SRP filtering. Therefore, FPs were high with LM filtering. The precision value was higher than the recall value using all methods, while ITD was higher at UAV 80 m than at UAV 120 m. Tree density was calculated based on ITD. The UAV tree density was higher than the field tree density (147 stems ha^−1^) at both flight altitudes due to the higher number of UAV treetop detections. Tree density was in the range of 155–190 stems ha^−1^ at UAV 80 m and 153–192 stems ha^−1^ at UAV 120 m.

### 4.3. Tree Height

**Figure 5:**
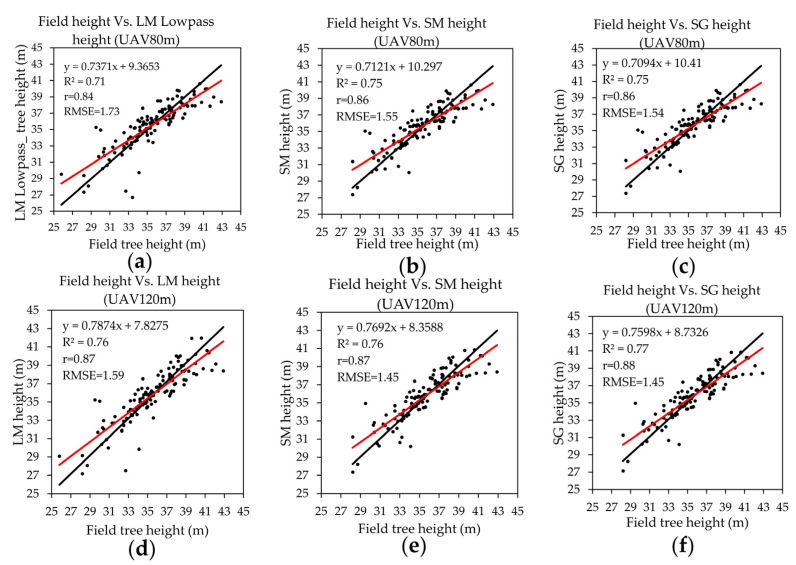
The scatter plots of field TH with UAV TH: (**a**) Field TH with LM lowpass filtering at UAV 80 m; (**b**) Field TH with SM filtering at UAV 80 m; (**c**) Field TH with SG filtering at UAV 80 m; (**d**) Field TH with LML filtering at UAV 120 m; (**e**) Field TH with SG filtering at UAV 120 m; (**f**) Field TH with SG filtering at UAV 120 m. Black lines represent the zero intercept of the trend line. Red lines represent the regression line of the data (black dots).

Table 5 shows the TH estimated from the LM algorithm and SRP. The LM and SRP results indicated that TH was slightly higher than the field TH at both flight altitudes. The maximum and minimum TH were slightly lower than the field TH minimum and maximum. Figure 5 shows the results of a simple linear regression of field TH with UAV TH. The R^2^ value for TH was slightly higher and had a lower RMSE at UAV 120 m than at UAV 80 m. Similarly, for the different filtering methods, the R^2^ value of LM filtering was lower than with SRP filtering, with a higher RMSE. At UAV 80 m, the R^2^ and RMSE values were 0.71 and 1.73 m, 0.75 and 1.55 m, and 0.75 and 1.54 m for LML filtering, SM filtering, and SG filtering, respectively. At UAV 120 m, the R^2^ and RMSE values were 0.76 and 1.59, 0.76 and 1.45 m, and 0.77 and 1.45 m for LML filtering, SM filtering, and SG filtering, respectively. 

### 4.4. CA and CC Percentages

**Figure 6:**
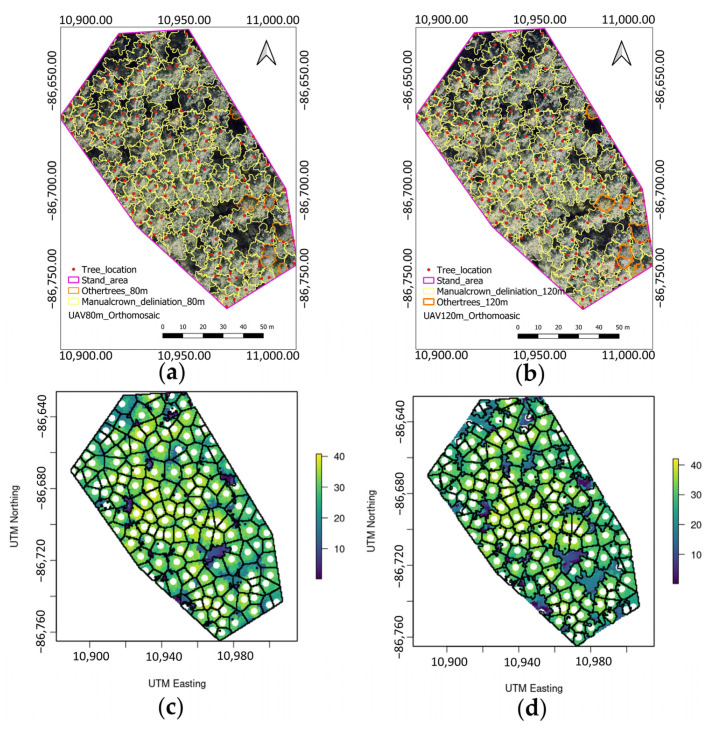
The results of the delineation of individual tree crowns: (**a**) Manual crown delineation at UAV 80 m with spatial positions of filed trees; (**b**) Manual crown delineation at UAV 120 m with spatial positions of filed trees; (**c**) SRP crown delineation at UAV 80 m with UAV treetops; (**d**) SRP crown delineation at UAV 120 m with UAV treetops. Yellow and orange lines in (**a**) and (**b**) represent the manual crown delineation of larch and other trees, respectively and red dots represent the spatial location of trees in the field. Black lines in (**c**) and (**d**) represent the SRP crown delineation of all trees and white dots represent the SRP detected UAV treetops.

Tree crowns, delineated manually and from the SRP, are presented in Figure 6. We manually delineated the tree crown of 136 larch (yellow) and seven other trees (orange) (Figure 6a,b). The SRP delineated the tree crowns automatically (Figure 6c,d). The manual CA (56.65 m^2^) of larch trees was lower than both the SRP mean (63.72 m^2^) and that obtained with Gaussian (63.83 m^2^) filtering at UAV 80 m. However, the SRP mean CA (56.44 m^2^) was slightly lower than the mean filtering CA (55.57 m^2^) and slightly higher than the Gaussian filtering CA (56.64 m^2^) at UAV 120 m. The maximum CA was higher with manual crown delineation than with the SRP at both UAV altitudes (Table 6). The manual CA delineation was more accurate than the SRP crown delineation due to the use of a high-resolution orthomosaic where the larch crown is easily visible. The CC percentage of larch with manual crown delineation (74.7%) was lower than that with SRP (92.0−92.7% at UAV 80 m and 78.2% at UAV 120 m). The total CC (including other species_estimated via manual crown delineation was 77.4% at both UAV altitudes, whereas the total CC obtained by SRP was 96% at UAV 80 m and 82.3% at UAV 120 m. 

**Figure 7:**
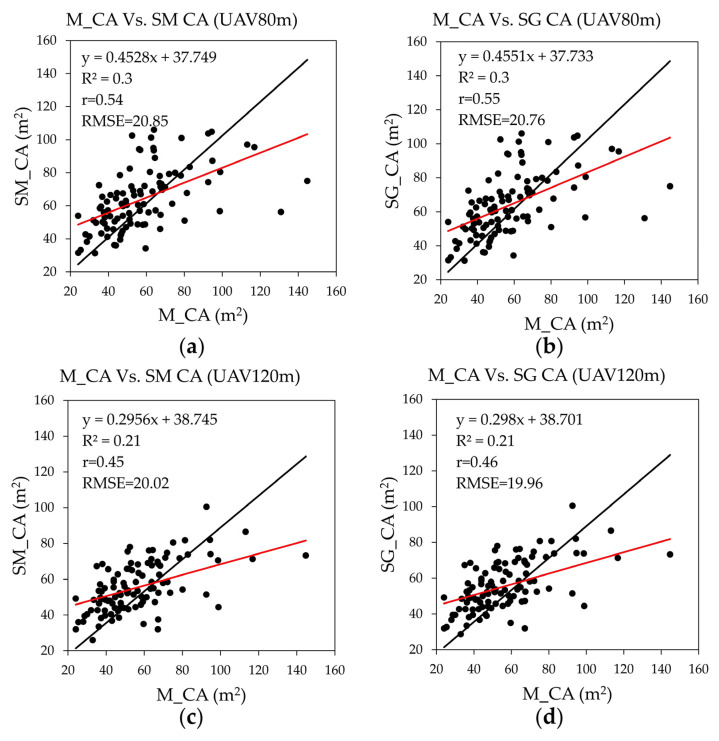
The scatter plots of manual CA with SRP CA: (**a**) Manual CA with SRP mean CA at UAV 80 m; (**b**) Manual CA with SG CA at UAV 80 m; (**c**) Manual CA with SM CA at UAV 120 m; (**d**) Manual CA with SG CA at UAV 120 m. Black lines represent the zero intercept of the trend line. Red lines represent the regression line of the data (black dots).

The results of a simple linear regression analysis are given in Figure 7. The R^2^ value for crown delineation was lower than that of the TH regression. The R^2^ value was higher at UAV 80 m than at UAV 120 m. At UAV 80 m, the R^2^ and RMSE values were 0.3 and 20.85 m^2^, and 0.30 and 20.76 m^2^ for mean and Gaussian filtering, respectively. At UAV 120 m, the R^2^ and RMSE values were 0.21 and 20.02 m^2^ and 0.21 and 19.96 m^2^ for mean and Gaussian filtering, respectively.

### 4.5. DBH, V, and CST

**Figure 8:**
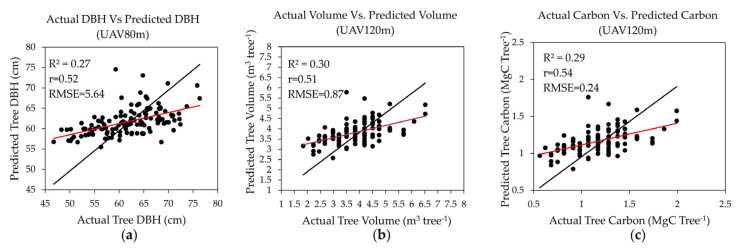
The scatter plots of DBH, V, and CST prediction with field estimated values: (**a**) Field DBH with Gaussian DBH at UAV 80 m; (**b**) Prediction of field V with SG volume at UAV 120 m; (**c**) Prediction of field CST with SG CST at UAV 120 m. Black lines represent the zero intercept of the trend line. Red lines represent the regression line of the data (black dots).

The results of the MLR model for tree DBH, V, and CST are given in Table 7. Figure 8 shows scatter plots of the predicted variables with field-estimated values. In the model, TH values derived from Gaussian filtering and manually digitalized CA were more accurate than those obtained using other filtering methods. DBH model had a lower R^2^ value (0.27) than the V and CST models, with an RMSE of 5.64 cm. Model R^2^ values for V and CST were 0.30 and 0.29, with an RMSE of 0.87 m^3^ tree^−1^ and 0.24 MgC tree^−1^, respectively. 

## 5. Discussion

### 5.1. Individual Tree Detection and Tree Density

For ITD, the careful selection of algorithms with suitable filtering/smoothing methods and window sizes influenced the accuracy of treetop detection [67,88]. The LM algorithm has strong potential to detect treetops [67], mainly in conifer plantations. We used the LM algorithm with different CHM filtering methods, radii, and window sizes. The F-score showed that the ITD was higher at UAV 80 m (0.87) than at UAV 120 m (0.83). Mohan et al. [82] showed that the F-score of the ITD in the canopy of a mixed conifer forest was 0.87. The ITD was higher in a conifer plantation than in a mixed broadleaf forest due to its homogenous structure. Our stand area was also a conifer plantation, but it was an old plantation with a canopy overlapping. Young et al. [48] reported that the accuracy of ITD and the resulting tree maps was generally maximized by collecting imagery at high altitude (120 m) with at least 90% image-to-image overlap in structurally complex mixed conifer forests where ITD ranged from 0.67 to 0.87, but the TH accuracy (R^2^ = 0.95) in their study was higher than in our study. In this study, when the tree density increased, the F-score decreased. We estimated tree density based on the ITD results. We tested the applicability of the SRP, an open-source application with a limited input size (30 megabytes) and a resolution of 0.5 m in the CHM [47]. When this model was larger and had a wider range of resolutions, ITD was more accurate. A detailed analysis is required to further increase the accuracy of the ITD. Using UAVs with hyperspectral images will increase the accuracy of ITD. Nevalainen et al. [89] obtained a high F-score of 0.93 with hyperspectral imagery for ITD in boreal forest.

### 5.2. Tree Height

In our study, TH accuracy was high, with a higher R^2^ and a lower RMSE for the UAV 120 m flight compared to the UAV 80 m flight. Pourreza et al. [81] reported that UAV data acquisition was not significantly different among three altitudes (25, 50, and 100 m) using a local network RTK system (NRTK), except for the mean values calculated at 100 m. They also obtained a positive and strong relationship between the measured and estimated TH (R^2^ > 0.99) at all three flight altitudes. The RMSE values for estimated TH at flight altitudes of 25, 50, and 100 m were 0.9%, 4.3%, and 10.2%, respectively, and the respective mean absolute error (MAE) values were 0.04, 0.21, and 0.52. Additionally, the findings indicated an underestimation of TH, which increased with increasing UAV flight altitude. Islami et al. [90] reported a high R^2^ value of 0.935 at a flight height of 100 m; this was higher than the values for the 80 m and 120 m flight altitudes in the present study, whereas the RMSE was lower at 100 m than at the other altitudes. Nasiri et al. [44] reported that the R^2^ and RMSE for TH, using a UAV and the LM algorithm, were 0.808 and 3.22 m, respectively. This RMSE was higher than our value (1.45–1.73 m), although it was calculated using a different method, while we obtained a lower R^2^ value (0.71–0.77). In a study of a cashew plantation, Mot et al. [80] reported that the highest R^2^ (0.60) was derived from a 50 m UAV flight, whereas the 200 m UAV flight only achieved an R^2^ of 0.50. They also revealed that their proposed method was only applicable to open terrain where the TH was <12 m due to a design limitation of the pipe meters (i.e., a straight tube to measure height). Because the cashew tree has a complex leaf, identifying the treetop was a challenge. 

The TH accuracy of conifer plantations in other studies was higher than in the present study due to the younger ages of their stands. Ota et al. [38] found that R^2^ and RMSE values for the mean TH from a CHM were in the ranges of 0.89–0.92 and 1.24–1.31, respectively, in an area dominated by 62-year-old plantations of evergreen conifer trees including Sugi and Hinoki. Krause et al. [11] reported a treetop detection rate > 80% using the LM algorithm in a 40-year-old conifer plantation, where the TH R^2^ value was 0.97–0.99 with an RMSE of 0.3–0.48 m. In general, our R^2^ value was low compared to studies in other conifer plantations. This may be due to the old-growth condition of the larch plantation in the present study, which had reached the stage of canopy overlapping. We extracted the TH based on the spatial position of the UAV tree location; hence, the TH accuracy depended on the accuracy of ITD. The accuracy of canopy detection was low due to the loss of apical dominance in old trees [91,92]. We found that some of the larch trees had a higher field TH than UAV TH. This was due to the edge effect of some trees in the field, which resulted in them being distributed in the high-height range of the CHM. Similarly, some trees had a lower field TH than UAV TH. This was due to some field trees being located outside of the high height range of the CHM, and there was no suitable height range in the CHM to match with the field TH. This may be due to the overlapping of lower trees by the treetop crown. For these reasons, the TH accuracy of the old larch plantation was lower than for other conifer plantations studied elsewhere. Therefore, the application of suitable algorithms and technologies that could penetrate or scan the vertical distribution of the tree canopy; for example, UAV LiDAR technology [93,94] in old plantations should be considered in future analyses.

### 5.3. Crown Delineation and CC Percentage

In our study, the mean manual CA SRP CA values were not statistically significantly different. The R^2^ value was higher at a lower altitude (UAV 80 m), with a slight change in the RMSE. Pourreza et al. [81] found that the mean crown diameter based on field measurements and UAV estimations at all flight altitudes were not statistically significantly different. The RMSEs for the estimated tree crown diameter at flight altitudes of 25, 50, and 100 m were 2.2%, 4.6%, and 10.7%, respectively. Additionally, Pourreza et al. [81] reported an underestimation of the crown diameter, which increased as the UAV flight altitude increased and also tended to increase with tree size. In our study, CA was overestimated by the SRP, and it decreased at higher altitudes due to the low pixel resolution and point density. Nasiri et al. [44] reported that the R^2^ and RMSE for crown diameter using the LM algorithm were 0.923 and 0.81 m (7.02%), respectively. The correlations for CA (0.45–0.55) were consistent with those of Moe et al. [28] who reported that the correlation between the UAV and manual CA values ranged from 0.45 to 0.57 in broadleaf tree species. He also reported low correlations between UAV and field-measured CA (0.23–0.44) values, but higher correlations were obtained for manual and field-measured CA (0.63–0.72) values. We analyzed the relationship between the manual and SRP CA values. The manual CA was lower than the SRP CA in many larch trees, resulting in an overestimation. This was because the SRP delineated a tree crown CHM that included the shadow area (i.e., the extended lower canopy of the tree crown) in the tree CA; however, CA was manually delineated based on the visual appearance of larch in the orthomosaic. Similarly, the manual CA had a higher value than the SRP CA in some trees, resulting in underestimation. This was because the SRP delineated the larch tree crown into two or more crowns. This explained the low accuracy of CA in our study. Manual crown delineation was more accurate in delineating multiple tree crowns due to the high-resolution orthomosaic. Therefore, various robust delineation approaches are needed to derive the CA of old larch plantations when the tree canopy contains multiple overlapping crowns.

### 5.4. Tree DBH, V, and CST

The values of our field stand parameters were high due to the plantation being 115 years old; plantations in other areas were much younger. The DBH of larch ranged from 10.9 to 23.7 cm at a 60-year-old Japanese larch plantation in central Japan [95]. Kita et al. [96] reported that TH, DBH, V, stand density, stand volume, and CST were in the ranges of 20.8–21.6 m, 21.4–27.7 cm, 0.393–0.587 m^3^ tree^−1^, 460–896 stem ha^−1^, 276–353 m^3^ ha^−1^, and 84.6–106.1 MgC ha^−1^, respectively, in a 31-year-old larch plantation. For the management of a single tree, the individual tree DBH is an important variable. However, the estimation of individual tree DBH from point-density-related remote sensing metrics is not significant. In this study, we developed a model using UAV-derived image metrics [28,97,98]. Yu et al. [97] stated that individual tree DBH estimated using the tree crown and height metrics was the best model. Chen et al. [98] reported that an individual tree V, estimated using LiDAR height and crown metrics, was the best model. Tree crown and TH measured in the field were used to develop the DBH models in previous studies [99,100]. In our study, we used UAV TH and CA metrics, as well as various filtering methods and the manual CA, to develop DBH, V, and CST models. The results of the DBH, V, and CST models also revealed that manual CA values, together with UAV TH values, could better estimate the DBH, V, and CST. Moe et al. [28] obtained R^2^ values of 0.32−0.47 using field CA and TH, while they were in the range of 0.4−0.56 using the manual CA and 99th percentile of TH in a conifer mixed broadleaf forest. Our R^2^ values (0.27−0.32) for the DBH, V, and CST models were closer to those of Moe et al. [28]. This may be due to the complexity of the old stand. However, a high R^2^ value could be obtained using LIDAR point clouds and other structural and textural UAV metrics.

### 5.5. Parameter Setting during the Photogrammetric Process

SfM and Multi-View Stereo (MVS) techniques were used in the UAV photogrammetry pipeline and these were processed in a fully automated way [101]. A 3D model of an object is built from the different positions of 2D photographs using the SfM technique [102]. The 3D model is created from the common features that were identified as matching points or key points in the 2D images through the SIFT (Scale Invariant Feature Transform) algorithms of SfM [103]. We used the same setting of Agisoft Metashape software (version 2.8) during the image alignment and other processes for both UAV altitudes of 80 m and 120 m to maintain consistency. Mousavi et al. [101] reported that tie point setting during the photogrammetric process will affect the accuracy of image orientation and outcome. Barazzetti [104] found that the improvement of precision is significant for a small number of points, whereas a huge number of 3D points does not provide significant improvement. Reprojection error, multiplicity, intersection angle, and a posteriori standard deviation are considered quality parameters during the point cloud extraction [105]. The aggregation of these quality metrics allows for the removal of low-quality tie points before refining the orientation results in a new adjustment. The lowest values for re-projection error and posteriori standard deviation, while the highest values for multiplicity and intersection angle showed high accuracy during the image alignment process. In this study, the mean reprojection error was higher for UAV 120 m (3.41 pix) than for UAV 80 m (2.84 pix). The authors will consider the algorithm of multi-criteria decision-making (MCDM) developed by Mousavi et al. [106] for future analysis to reduce the reprojection error.

However, our RMSE was 0.000553 m and 0.331 pix for UAV 80 m, and 0.000481 m and 0.367 pix for UAV 120 m, during image alignment when two GCPs were added in Agisoft Metashape, confirming the accuracy of the measurements. Tavasci et al. [79] obtained an RMSE of 0.06 m with seven GCPs and a GSD of 0.03 m using RTK GNSS, and they confirmed the good quality of the measurement. Izere [107] stated that UAV Phantom 4 RTK M300 with RTK GNSS enabled high accuracy on plant height estimation without GCPs. As a result, it has been suggested that this accurate positioning information can serve as a viable alternative to the traditional use of GCPs for georeferencing photogrammetric models [108,109,110]. Tahar [111] found that the error range was decreased after using seven or more GCPs in 150 ha. Kalacska et al. [112] concluded that where repeatability and adherence to a high level of accuracy are needed, only RTK and PPK systems should be used without GCPs. Stott et al. [113] stated that using no GCPs and 5 GCPs with 3300 independent spatially distributed RTK-GNSS surveyed checkpoints gave an RMSE of 0.066 and 0.072 m, respectively. Štroner et al. [114] combined DJI Phantom 4 RTK with RTK-GNSS methods, giving the best results for both the vertical and horizontal components, but using a small number of GCPs (at least one) or quality camera pre-calibration is applicable where the terrain is difficult for SfM evaluation. Martínez-Carricondo et al. [115] minimized the altimetry errors by placing 1.7 GCPs around the edge of the study area. Yu et al. [116] reported that 12 GCPs and 18 GCPs were optimal for 7–39 ha and 342 ha, respectively.

GCPs rigorously incorporated in the adjustment remain mandatory to control network deformation. Accuracy is also dependent on the software [58]. We therefore used two highly accurate GCPs using RTK GNSS and calibrated images for the photogrammetric process using Agisoft Metashape. Additionally, Swayze et al. [43] stated that Agisoft Metashape-estimated horizontal alignment error was not significantly different with the acquisition altitude of UAV flight. We also found that the image resolution was reduced when the altitude increased (3.25 cm/pix at UAV 80 m and 5.39 cm/pix at UAV 120 m), this also played the accuracy of the outcome. Frey et al. [117] stated that the influence of flight parameters on TH and crown diameter was studied; however, there was a knowledge gap on other stand parameters. They also reported that the accuracy of TH was high for all UAV flight parameters; however, the accuracy of DBH was high for lower altitudes. Image alignment and positional accuracy of the point clouds are the sources of error in extracting individual tree locations and DBH. However, they used a 4 m buffer during the tree-matching process to reduce spatial positional errors [43]. In this study, the accuracy of ITD and TH was obtained without buffer.

## 6. Conclusions

Many studies have been conducted in old-growth forests and plantations using various remote sensing technologies, but to the best of our knowledge, stand parameters have not been estimated using UAV technology in an old Japanese larch plantation. The old larch plantation in this study had mean TH, DBH, BA, V, and CST values of 35.2 m tree^−1^, 60.9 cm tree^−1^, 0.3 m^2^ tree^−1^, 3.76 m^3^ tree^−1^, and 1.15 MgC tree^−1^, respectively, while tree density, stand V, and stand CST were 154 stem ha^−1^, 543 m^3^ ha^−1^, and 168 MgC ha^−1^, respectively. The CHM was generated using UAV DSM and LiDAR DTM to ensure the accuracy of the extracted stand parameters. From the UAV photogrammetry results, the accuracy of ITD and TH was higher than that of the CA, DBH, V, and CST. For the different UAV flying altitudes, the accuracy of ITD, CA, and DBH was highest at UAV 80 m, whereas the accuracy of TH, V, and CST was highest at UAV 120 m. 

Increasing the search radius and window size improved the ITD rate. When comparing the different filtering methods, the accuracy of TH was highest for both mean and Gaussian filtering, while for CA it was highest for Gaussian filtering. Comparatively, higher accuracy was obtained with Gaussian and mean filtering. Only the high-resolution UAV orthomosaic enabled highly accurate manual crown delineation. For DBH, V, and CST estimation, the best model was obtained after fitting with the metrics of manually digitalized CA and UAV TH. We found that the accuracy of the stand parameters depended on the altitude of the UAV and the filtering method used. Therefore, forest managers should be aware that the estimation of stand parameters depends on the UAV’s flight altitude and filtering methods. In general, varying the flying altitude and related algorithms, together with the use of various filtering methods in old Japanese larch plantations, may improve stand parameter estimation. Therefore, as in other conifer plantations, we speculate that there will be variations in the estimated value. However, a detailed investigation is needed for other old conifer plantations that are distributed with different formats of stand structure such as shape of the tree crown, distribution of crown area, acuteness of canopy top, and number of branches. Future studies should focus on refining these methods, exploring the potential of other algorithms and techniques, using high-resolution hyperspectral imagery for more accurate and efficient tree detection, and stand parameter estimation using UAV photogrammetry.

## Figures and Tables

**Table 1:** Summary statistics of the field data.

Field Parameter	Unit	Mean	SD *	Range
Tree height (TH)	m tree^−1^	35.20	3.27	25.80–42.90
Tree diameter (DBH)	cm tree^−1^	60.94	7.14	45.45–79.93
Basal area (BA)	m^2^ tree^−1^	0.30	0.07	0.16–0.50
Stem volume (V)	m^3^ tree^−1^	3.76	1.10	1.84–7.42
Carbon stock (CST)	MgC tree^−1^	1.15	0.34	0.56–2.27
Tree density	stems ha^–1^	147		

* SD—standard deviation.

**Table 2:** Specifications and parameter setting of the UAV for imagery collection.

UAV Parameter	Setting
Model	DJI Matrice 300 RTK (Da-Jiang Innovations, Shenzhen, China)
Camera model	DJI Zenmuse P1 RGB (Da-Jiang Innovations, Shenzhen, China)
Lens specifications *	Sensor Dimensions: 35.000 mm × 23.328 mm Resolution: 8192 × 5460 Focal Length: 35 mm Pixel Size: 4.39 × 4.39 μm
Flight altitude	80 m and 120 m
Front overlap	90%
Side overlap	90%
Flight time	30 min
Flight take-off speed	15 m/s
Average Flight speed—80 m	5 m/s
Average Flight speed—120 m	7 m/s
Ground Sampling Distance—80 m	1.00 cm/pixel
Ground Sampling Distance—120 m	1.51 cm/pixel

* Processing report of Agisoft Metashape.

**Table 3:** Individual tree detection (ITD) by a combination of filtering, search radius, and window sizes at two flight altitudes of UAV 80 m and UAV 120 m.

UAV Altitude	Filtering Method *	Search Radius/Sigma (m)	Window Size (m)/Search Method	UAV Treetop **
UAV 80 m	LML	1	circle	304
		2	circle	182
		3	circle	135
	LMG	1	circle	316
		2	circle	224
		3	circle	184
		4	circle	182
		5	circle	182
	SM		FWS: 3 × 3, SWS: 3 × 3	242
			FWS: 5 × 5, SWS: 3 × 3	151
	SG	1	FWS: 3 × 3	258
		2	FWS: 3 × 3	245
		3	FWS: 3 × 3	244
		1	FWS: 5 × 5	154
		2	FWS: 5 × 5	152
		3	FWS: 5 × 5	153
UAV 120 m	LML	1	circle	300
		2	circle	182
		3	circle	131
	LMG	1	circle	317
		2	circle	226
		3	circle	190
		4	circle	187
		5	circle	187
	SM		FWS: 3 × 3, SWS: 3 × 3	243
			FWS: 5 × 5, SWS: 3 × 3	149
	SG	1	FWS: 3 × 3	249
		2	FWS: 3 × 3	244
		1	FWS: 5 × 5	150
		2	FWS: 5 × 5	149

* LML—local maxima lowpass filtering; LMG—local maxima Gaussian filtering; SM—shiny mean filtering; SG—shiny Gaussian filtering; ** UAV treetop—detected number of treetops.

**Table 4:** The results of ITD (UAV treetop) with tree density, precision, recall, and F-score (TP, FP, and FN) by different filtering methods at two UAV flying altitudes.

UAV Altitude	Filtering Method *	UAV Treetop	Tree Density	TP	FP	FN	Precision	Recall	F-Score
UAV 80 m	LML2	176	189.09	124	52	12	0.91	0.70	0.79
	SM5	144	154.71	122	22	14	0.90	0.85	0.87
	SG2	145	155.78	122	23	14	0.90	0.84	0.87
UAV 120 m	LML2	178	191.24	119	59	17	0.88	0.67	0.76
	SM5	142	152.56	116	26	20	0.85	0.82	0.83
	SG2	142	152.56	115	27	21	0.85	0.81	0.83

* LML2—local maxima lowpass filtering at search radius 2 m; SM5—shiny mean filtering at search radius 5 m; SG2—shiny Gaussian filtering at search radius 2 m.

**Table 5:** The results of mean TH with maximum and minimum values for different filtering methods at two UAV flying altitudes with field TH.

Field and UAV Altitude	Filtering Method *	Mean TH ± SD (m)	Maximum	Minimum
Field	Field TH (n = 136)	35.23 ± 3.27	42.90	25.80
UAV 80 m	LML2 (n = 136)	35.49 ± 2.81	40.62	26.71
	SM5 (n = 123)	35.63 ± 2.55	40.62	27.40
	SG2 (n = 122)	35.65 ± 2.52	40.62	27.40
UAV 120 m	LML2 (n = 125)	35.76 ± 2.89	41.97	27.18
	SM5 (n = 123)	35.08 ± 2.63	40.85	27.37
	SG2 (n = 122)	35.81 ± 2.62	40.86	27.15

* LML2—local maxima lowpass filtering at search radius 2 m; SM5—shiny mean filtering at search radius 5 m; SG2—shiny Gaussian filtering at search radius 2 m; n—number of trees.

**Table 6:** The results of CA with maximum and minimum values and CC for different filtering methods at two UAV flying altitudes.

UAV Altitude	Filtering Method *	Mean TH ± SD (m)	Maximum	Minimum	CC Larch (%)	CC All (%)
UAV 80 m	Manual CA (n = 136)	56.65 ± 21.27	144.76	23.89	74.74	77.44
	SM5 (n = 98)	63.72 ± 18.73	106.00	31.25	92.02	96.05
	SG2 (n = 98)	63.83 ± 18.65	106.00	31.25	92.77	96.06
UAV 120 m	Manual CA (n = 136)	56.44 ± 21.24	144.76	23.89	74.74	77.44
	SM5 (n = 106)	55.57 ± 14.33	100.50	19.50	78.72	82.29
	SG2 (n = 95)	56.64 ± 14.29	100.50	19.50	78.72	82.29

* LML2—local maxima lowpass filtering at search radius 2 m; SM5—shiny mean filtering at search radius 5 m; SG2—shiny Gaussian filtering at search radius 2 m.

**Table 7:** The results of the MLR model for tree DBH, V, and CST for two flight altitudes.

UAV Altitude	Dependent Variable (Unit)	Independent Variables **	Selected Model	Parameter Estimates	R^2^	RMSE
UAV 80 m	DBH (cm)	SG_TH, ND, M_CA, C_peri	Intercept	45.02948 ***	0.27	5.64
			SG_TH	0.20821 *		
			M_CA	0.16068 ***		
UAV 120 m	V (m^3^ tree^−1^)	SG_TH, M_CA, ND, SG_CA	Intercept	−0.773327 ***	0.30	0.87
			SG_TH	0.086222 *		
			M_CA	0.025442 ***		
	C (MgC tree^−1^)	SG_TH, M_CA, ND, SG_CA	Intercept	−0.229230 ***	0.29	0.24
			SG_TH	0.026223 *		
			M_CA	0.007716 ***		

** M_CA—manual crown area; F_TH—field tree height; C_peri—the perimeter of a tree crown in m; ND—near distance, is the shortest distance of a tree that is near to the target tree or Euclidean distance from each cell in the raster to the closest source in m; SM_TH—SRP mean tree height; SG_TH—SRP Gaussian tree height; significance code—*** *p* < 0.001, * *p* < 0.05.

## Data Availability

The field and UAV datasets presented in this study are available on request from the corresponding author.

## Appendix A

**Table A1:** Parameter setting of UAV image processing.

Photogrammetric Process	Parameters
Image alignment	Accuracy: medium
	Pair selection: Reference
	Key points: 40,000
	Tie points: 1000
Guided marker positioning	4
Camera optimization parameters	F, b1, b2, cx, cy, k1–k4, p1, p2
Building dense cloud	Quality: medium
	Depth filtering: Mild
	Quality: medium
	Depth filtering: mild
Building mesh	Surface type: medium
	Source data: Dense cloud
	Interpolation: Disabled
	Face count: high
Building DEM	Type: Geographic
	Source data: Dense cloud
	Interpolation: enabled
Building orthomosaic	Type: Geographic
	Surface: DEM
	Blending mode: Mosaic
	Enable hole filling

**Table A2:** Processing time of UAV imagery in Agisoft Metashape (version 1.8.4.)

Photogrammetric Process	Setting	UAV 80 m	UAV 120 m
Image alignment	Matching time	15 min 34 s	4 min 9 s
	Alignment time	25 min 55 s	3 min 34 s
Camera optimization	Optimization time	1 min 13 s	13 s
Building dense cloud	Processing time	1 h 1 min	18 min 5 s
	Generation time	2 h 7 min	18 min 5 s
	Depth map and reconstruct	1 h 30 min	1 h 7 min
Building DEM	Processing time	2 min 6 s	15 s
Building Orthomosaic	Processing time	1 h 28 min	1 h 5 min
Systems used	Software: Agsoft Metashape Professional Software version: 1.8.4 build 14856 OS: Windows 64 bit RAM: 31.71 GB CPU: 11th Gen Inter(R) Core (TM) i7-11800H @2.30 GHz GPU: NVIDIA GeForce RTX 3050 Ti Laptop GPU		

**Table A3:** Results of drone-image processing.

UAV Attribute	UAV 80 m	UAV 120 m
Acquired images	1257	372
Flying altitude	80 m	120 m
Point density	931 points/m^2^	328 points/m^2^
Pixel resolution	3.25 cm/pix	5.39 cm/pix
Image resolution	8192 × 5460 pix	8192 × 5460 pix
Ground resolution	0.813 cm/pix	1.35 cm/pix
Tie points	1,109,968	483,325
Projections	3,707,621	1,030,596
Mean reprojection error (pixel)	2.84 pix	3.04 pix
Total error with GCPs	0.331 pix	0.367 pix
Dense clouds	136,581,158	74,639,588
Coordinate system	JGD2000 Japan–19 zone XII/GSIGEO 2000 geoid	
